# The zinc finger protein ZFP36L2 inhibits flavivirus infection via the 5′-3′ XRN1-mediated RNA decay pathway in the replication complexes

**DOI:** 10.1186/s12929-025-01122-0

**Published:** 2025-02-20

**Authors:** Ren-Jye Lin, Li-Hsiung Lin, Zih-Ping Chen, Bing-Cheng Liu, Pin-Chen Ko, Ching-Len Liao

**Affiliations:** 1 National Mosquito-Borne Diseases Control Research Center, National Health Research Institute, Taipei, Taiwan; 2 National Institute of Infectious Diseases and Vaccinology, National Health Research Institutes, Miaoli, Taiwan; 3 Institute of Preventive Medicine, National Defense Medical Center, New Taipei, Taiwan

**Keywords:** CCCH-type zinc finger protein, ZFP36L2, 5′- 3′ XRN1, Antiviral mechanism, Processing bodies, Replication complexes, RNA decay pathway

## Abstract

**Background:**

The zinc finger protein 36-like (ZFP36L) family is a CCCH-type group consisting of RNA-binding proteins, i.e., ZFP36L1 and ZFP36L2, which regulate cellular mRNA through the RNA decay pathway. ZFP36L1 combats flavivirus infections through the 5′-3′ XRN1 and 3′-5′ RNA exosome decay pathways. The present study clarified the role of human ZFP36L2 in the defense response of the host against flavivirus infection.

**Methods:**

Cell lines with overexpression or knockdown of ZFP36L2 were established using lentiviral vectors carrying genes for overexpression and short-hairpin RNA targeting specific genes, respectively. A plaque assay was employed to determine the viral titer. Immunofluorescence and real-time quantitative polymerase chain reaction were used to measure the viral RNA levels. The in vitro-transcribed RNA transcript derived from a replication-dead Japanese encephalitis virus (JEV) replicon containing the renilla luciferase reporter gene (*J-R2A-NS5mt*) was used to assess the stability of the flavivirus RNA. An RNA immunoprecipitation assay was used to detect the protein–RNA binding ability. Confocal microscopic images were captured to analyze protein colocalization.

**Results:**

ZFP36L2 served as an innate host defender against JEV and dengue virus. ZFP36L2 inhibited flavivirus infection solely through the 5′-3′ XRN1 RNA decay pathway, whereas ZFP36L1 inhibited JEV infection via the 5′-3′ XRN1 and 3′-5′ RNA exosome RNA decay pathways. The direct binding between viral RNA and ZFP36L2 via its CCCH-type zinc finger motifs facilitated the degradation of flavivirus RNA mediated by 5′-3′ XRN1. Furthermore, ZFP36L2 was localized in processing bodies (PBs), which participate in the 5′-3′ XRN1-mediated RNA decay pathway. Nonetheless, the disruption of PBs did not affect the antiviral activity of ZFP36L2, suggesting that its localization is not essential for the function of the protein. Interestingly, the colocalization of ZFP36L2 and XRN1 with viral RNA and NS3 revealed that the antiviral activity of ZFP36L2 occurred within the replication complexes (RCs).

**Conclusions:**

In summary, ZFP36L2 bound to and degraded viral RNA through the XRN1-mediated RNA decay pathway in the RCs, thereby inhibiting flavivirus replication. These findings provide valuable insights into the diverse antiviral mechanisms of the ZFP36-like family of proteins in the innate immune response against flavivirus infection.

**Supplementary Information:**

The online version contains supplementary material available at 10.1186/s12929-025-01122-0.

## Background

The restriction of the accumulation of viral RNA transcripts by the cellular RNA decay machinery plays a crucial role in antiviral defense in infected cells, as exemplified by the system formed by interferon (IFN)-induced 2′,5′-oligoadenylate synthetase (OAS) and its downstream effector RNase L (RNase L) [1, 2]. A zinc-finger antiviral protein (ZAP), i.e., zinc-finger CCCH-type antiviral 1 (ZC3HAV1), was the first member identified in the CCCH-type family and was shown to afford a potent defense against specific RNA viruses [3]. It combats viral infections via direct binding to viral RNA and the recruitment of the cellular mRNA decay machinery, including tripartite motif containing 25 (TRIM25), KH and NYN domain-containing (KHNYN), p72 RNA helicase, the 5′-3′ exoribonuclease XRN1, and the 3′-5′ RNA exosome complex, to degrade the viral RNA transcripts [4–7]. Tetrachlorodibenzo-*p*-dioxin (TCDD)-inducible poly(ADP-ribose) polymerase (TIPARP) has antiviral functions similar to those of ZAP. It binds viral RNA through the ZF motifs and degrades it via the 3′-5′ RNA exosome complex, thus inhibiting Sindbis viral replication [8]. A recent study showed that the potent antiviral activity of ZFP36L1 against Japanese encephalitis virus (JEV) and dengue virus (DENV) infection is caused by direct binding to the genome and recruitment of both the 5′-3′ and 3′-5′ XRN1 RNA exosome-mediated RNA decay machinery, for viral degradation [9].

The human zinc finger protein ZFP36L2, a CCCH-type, belongs to the ZFP36 family, which includes two paralogs, ZFP36 and ZFP36L1 [10]. Moreover, it is known as TIS11D/BRF-2/ERF-2ZFP36L2, is characterized by tandem CCCH-type ZF motifs, and functions as an RNA-binding protein (RBP) that interacts with the AU-rich element (ARE) in the 3′-untranslated region (UTR) of the messenger RNA (mRNA) of the target gene. This interaction destabilizes the target mRNAs, thereby interfering with the post-transcriptional regulation of gene expression [11–14]. In addition, as a multifunctional protein involved in various biological and physiological processes, ZFP36L2 stabilizes the low-density lipoprotein receptor (*LDLR*) mRNA [11], is associated with female fertility and early embryonic development [14], is critical for definitive hematopoiesis during development in the mouse [15], contributes to thymocyte development, prevents the pathogenesis of T-cell acute lymphoblastic leukemia (T-ALL) [16], and represses the translation of pre-formed cytokine-encoding mRNAs in memory T cells [17]. Moreover, ZFP36L2 has been implicated in cell-cycle arrest [18], tumor necrosis factor-alpha (TNF-α) mRNA-destabilizing capability [13], B-cell development [19], skeletal muscle myogenesis [20], cancer progression [21], the regulation of mitochondrial fusion/fission during myocardial ischemia/reperfusion injury [22], and the negative regulation of TNF-α-induced CXCL8 (also known as interleukin 8, IL-8) in dermal fibroblasts [23]. These diverse roles highlight the significance of ZFP36L2 in various physiological and pathological processes.

Cytoplasmic RNA granules, such as processing bodies (P-bodies; PBs) and stress granules (SGs), are critical cellular structures that regulate post-transcriptional mRNA metabolism and gene expression. PBs serve as sites for mRNA decay or storage, whereas SGs play a role in translational repression [24–26]. Emerging evidence supports the contention that the induction of PBs and SGs in the host promotes the antiviral response by sequestering viral RNA transcripts for sequential degradation and repression of translation, respectively [27–30]. PBs carry various components of the mRNA-degradation machinery, including XRN1, which is involved in the 5′-3′ RNA decay pathway [31, 32]. Moreover, XRN1 is an antiviral host factor that degrades the viral RNA transcripts by removing nucleotides from the 5′ end of the HCV RNA, thus restricting the replication of various positive-sense RNA viruses [33, 34]. In conjunction with the decapping enzymes DCP1/2, XRN1 exerts an antiviral activity against Newcastle disease virus and encephalomyocarditis virus (EMCV), which are cytoplasmic RNA viruses [35].

JEV and DENV are the major mosquito-borne pathogens of the *Flavivirus* genus, causing numerous infections and deaths globally [36, 37]. Flaviviruses are enveloped and carry a single-stranded, positive-sense RNA genome consisting of a 5′-cap structure but lacking a 3′-poly(A) tail. Furthermore, the viral genome encodes a single polyprotein that is processed by both cellular and viral proteases into three structural [including the capsid (C), precursor membrane (prM), and envelope (E)] proteins that are essential for virion assembly; whereas seven nonstructural (NS) proteins (i.e., NS1, NS2A, NS2B, NS3, NS4A, NS4B, and NS5) are involved in the RNA replication of the viruses. Flavivirus replication occurs on membrane structures derived from the endoplasmic reticulum (ER) network in which the intracellular membranes are modified to encompass the RNA-replication machinery and form replication complexes (RCs) [38, 39].

In a previous study, ZFP36L1 was shown to provide an innate defense against flavivirus via the 5′-3′ XRN1 and 3′-5′ RNA exosome-mediated RNA decay pathways [9]. Nevertheless, the mechanisms underlying the action of ZFP36L2 against flaviviruses remain unknown. Here, we demonstrated the potent antiviral activity of human ZFP36L2 against JEV and DENV infections. The underlying antiviral mechanism involved the direct binding of ZFP36L2 to the flavivirus RNA genome and the subsequent degradation of the viral RNA transcripts by recruiting the 5′-3′ XRN1-mediated RNA decay pathway. Furthermore, we demonstrated that ZFP36L2 functioned as a component of PBs and SGs; however, the antiviral activity of ZFP36L2 mediated by XRN1 occurred in viral RCs and was independent of its localization in PBs. Thus, the members of the zinc finger protein 36-like family, specifically ZFP36L1 and ZFP36L2, seem to be RBPs that exert antiviral effects through diverse pathways.

## Methods

### Viruses, cell lines, and chemicals

The JEV PR-9 (GenBank accession: AF014161) [40] and DENV-2 PL046 (GenBank accession: KJ734727) [41] strains were propagated in mosquito C6/36 cells (ATCC, CRL-1660) cultured in RPMI-1640 medium containing 5% fetal bovine serum (FBS). Using the plaque assay, the JEV and DENV titer was determined in baby hamster kidney BHK-21 cells (ATCC, CCL-10) cultured in RPMI-1640 medium supplemented with 5% FBS, 2 mM l-glutamine, and 1% penicillin–streptomycin (P/S). Human lung carcinoma A549 cells (ATCC, CCL-185) were cultured in F-12 medium in the presence of 10% FBS, 2 mM l-glutamine, and 1% P/S. The tetracycline (Tet)-regulated human embryonic kidney 293 (HEK-293) cell line T-REx-293 (Invitrogen) was cultured in Dulbecco’s Modified Eagle’s Medium (DMEM) containing 10% FBS and 5 μg/mL blasticidin. T-REx-293 cells expressing HA-tagged ZFP36L1 and ZFP36L2 were established as described previously [42] and cultured in DMEM containing 10% tetracycline-free FBS, 5 μg/mL blasticidin, and 250 μg/mL hygromycin. HEK293T/17 cells (ATCC, CRL-11268) and African green monkey kidney COS-7 cell line (ATCC, CRL-1651) were cultured in DMEM containing 10% FBS, 2 mM l-glutamine, and 1% P/S. Lipofectamine 2000 (Invitrogen) was used for DNA and RNA transfection. Doxycycline (Dox) was obtained from Clontech; and hygromycin, blasticidin, and puromycin were purchased from InvivoGen.

### Plasmids, lentiviruses, and stable shRNA cell lines

The cDNA fragment encoding human ZFP36L2 (GenBank accession: NM_006887) was cloned into the pcDNA5/TO (Invitrogen) and the self-inactivating lentiviral (pSIN vector) vectors with an N-terminal HA-tag, as described previously [42]. The plasmid expressing mCherry-fused ZFP36L2 protein was constructed using the pmCherry-C3 vector (Addgene). The ZF mutant of ZFP36L2 (C174R/C212R) was generated by mutagenesis [43] using C174R (5′-CGTGCAAGTACGGCGAAAAGCGCCAGTTCGCGCATGGCTTC-3′) and C212R (5′-CCCTATGGGCCGCGCCGCCACTTCATCCACAACGCGG-3′) (mutation underlined) primers. The recombinant plasmids pT7-EGFP-C1-XRN1, pT7-EGFP-C1-G3BP1, and pT7-EGFP-C1-DCP1a, expressing the XRN1, G3BP1, and Dcp1a proteins, respectively, fused to an enhanced green fluorescent protein (EGFP) were procured from Addgene. The lentiviral vectors carried a short-hairpin RNA (shRNA) targeting specific genes, including human *ZFP36L2* (#1: TRCN0000429458, 5′-ATCAACTCCACGCGCTACAAG-3′; and #2: TRCN0000013625, 5′-CTTCTTGTGCAAGACAGAGAA-3′), human *XRN1* (TRCN0000296739, 5′-GTTACTCACAGGTCGTAAATA-3′), human *EXOSC5* (TRCN0000050621, 5′-GTGAAGGTCAGCAAAGAGATT-3′), human *eIF4E-T* (#1: TRCN0000153716, 5′-CGCCAAAGTTATCAGTGTAGA-3′ and #2: TRCN0000427854, 5′-GGATCACCGTCTTAGCGATAAT-3′), and *LacZ* (TRCN0000072223, 5′-CGCGATCGTAATCACCCGAGT-3′). The lentivirus used for the expression of the proteins or shRNAs was prepared as described previously [42]. The knockdown cells were established by transduction with lentiviruses expressing specific shRNAs for 24 h, and selected using puromycin (5 μg/mL) for 72 h [42].

### Antibodies

The mouse monoclonal anti-JEV NS3 and anti-DENV NS3 antibodies were established previously [41, 44]. The commercial antibodies used in this study were as follows: anti-actin (NB600-501) and anti-XRN1 (NB500-191), from Novus Biologicals; anti-HA (C29F4) and anti-BRF1/2 (#2119) against ZFP36L1, from Cell Signaling Technology; anti-TIS11D (sc-365908) to detect ZFP36L2, from Santa Cruz Biotechnology; anti-dsRNA (J2), from Scicons; anti-EXOSC5 (ab168804), anti-Dcp1a (ab47811), Alexa Fluor 647 anti-Calreticulin antibody (ab196159) and anti-eIF4E-T (ab95030), from Abcam; and anti-G3BP (#61126), from BD Biosciences.

### Western blot analysis

The cell lysates were analyzed by sodium dodecyl sulfate polyacrylamide gel electrophoresis (SDS–PAGE) followed by western blot analysis [45]. The data were quantified using the ImageJ software.

### RNA immunoprecipitation assay

Briefly, HEK-293 cells were infected with DENV-2 or JEV [multiplicity of infection (MOI) = 1] for 6 h, and then transfected with plasmids expressing EGFP, HA-ZFP36L2 (wild-type), and HA-ZFP36L2 (mutant) for 18 h, respectively. Subsequently, the cells were lysed in RIPA buffer [10 mM Tris–HCl (pH 7.5), 150 mM NaCl, 5 mM EDTA, 0.1% SDS, 1% Triton X-100, and 1% sodium deoxycholate] containing a protease inhibitor cocktail (Roche) and RNasin (Promega). The lysate was incubated with the EZview Red Anti-HA Affinity Gel (Sigma-Aldrich) at 4 °C for 16 h. The RNA that was pulled down in the RNA–protein complexes was extracted using the RNeasy Mini Kit (Qiagen) and analyzed by RT-PCR using JEV and DENV 3′-UTR-specific primers [46].

### Real-time quantitative RT-PCR (RT-qPCR)

Total RNA was extracted using the RNeasy Mini Kit and reverse transcribed using the SuperScript III First-Strand Synthesis kit and random hexamer primers (Invitrogen). Subsequently, RT-qPCR was carried out using the TaqMan assay on an ABI-Prism 7900HT sequence detection system. The relative RNA levels of the target gene were measured using the comparative threshold cycle (Δ*C*_*T*_) method and normalized to those of the *GAPDH* housekeeping gene. The TaqMan Master Mix with UNG (Applied Biosystems), commercial probes for human *GAPDH* (Hs02758991) and firefly luciferase (Mr03987587) (Applied Biosystems), and JEV viral RNA primers were as listed previously [47].

### In vitro transcription

The SP6-JR2A NS5mt/pBR322 plasmid, which is an RNA-dependent RNA polymerase (RdRP)-dead JEV replicon DNA with the *SP6* promoter and the upstream sequence of the renilla luciferase (*Rluc*) gene flanked by the JEV 5′ UTR [48]; and the pGL3 luciferase reporter vector containing the firefly luciferase (*Fluc*) gene were as described previously [46]. Moreover, 5′-capped RNA transcripts were synthesized using the cap analog [m7G(5′)ppp(5′)G] (Ambion) and the RiboMAX Large Scale RNA Production System-SP6 (Promega), according to the manufacturer’s instructions, then purified using the Direct-zol RNA kit (Zymo Research). The RNA quality was assessed by agarose electrophoresis.

### Luciferase assay

Briefly, T-REx-293 cells expressing HA-ZFP36L1 and HA-ZFP36L2 were cultured with or without Dox (1 μg/mL) for 6 h, followed by cotransfection with 5′-capped renilla luciferase containing the JR2A-NS5mt replicon RNA and control firefly luciferase RNA. At 24 h post-transfection, the luciferase activity was measured in the cell lysates using the dual-luciferase assay (Promega). The relative luciferase activity (Rluc/Fluc) was calculated based on the ratio of renilla and firefly luciferase activities.

### Immunofluorescence assay (IFA)

A549 cells were transduced with the lentiviral vectors overexpressing the HA-ZFP36L1, HA-ZFP36L2, or control EGFP proteins for 72 h, and then infected with JEV (MOI = 5) for 24 h. Subsequently, the cells were fixed with 4% formaldehyde, permeabilized with 0.5% Triton X-100, and blocked with 5% skimmed milk at room temperature for 30 min. Next, mouse anti-dsRNA and Alexa Fluor 568 goat anti-mouse antibodies (Molecular Probes) were used to detect viral dsRNA, whereas HA-ZFP36L1 and HA-ZFP36L2 expression was detected using rabbit anti-HA and Alexa Fluor 488 goat anti-rabbit (Molecular Probes) antibodies. The nuclei were stained with 4′,6′-diamidino-2-phenylindole (DAPI; Molecular Probes), and images were captured using a fluorescence microscope (IX73; Olympus).

### Confocal microscopy

Cells were grown on chamber slides (ibidi), then fixed with 4% paraformaldehyde at room temperature for 20 min, permeabilized in PBS containing 0.1% Triton X-100, and blocked with 1% bovine serum albumin (Sigma–Aldrich) in PBS. Subsequently, the cells were mounted with the ProLong Gold Antifade reagent (Invitrogen) and examined under a confocal laser scanning microscope. During the analysis of the subcellular localization of the ZFP36L2 and XRN1 proteins, the viral RNA and JEV NS3 protein were detected using anti-dsRNA (J2; Scicons) and anti-JEV NS3 antibodies, respectively. Subsequently, the viral RNA and protein components were labeled with secondary antibodies conjugated with Alexa Fluor 647 (Invitrogen). The ER was labeled using an Alexa Fluor 647-conjugated anti-calreticulin antibody (Abcam). The nuclei were stained with DAPI (Molecular Probes), and the images were collected under a confocal microscope (Stellaris 8; Leica). Colocalization was measured and quantified by the Imaris software (Imaris Version 9.5.0, Bitplane AG, Switzerland).

### Statistical analysis

Two-tailed Student’s *t*-test was used to determine the statistical significance of the differences observed between the two groups, and data of triplicate samples (n = 3) are presented as the mean ± standard deviation (SD). *P* ≤ 0.05 indicated a statistically significant difference. * P ≤ 0.05; ** P ≤ 0.01; *** P ≤ 0.001; NS, not significant.

## Results

### Overexpression of the human ZFP36L1 and ZFP36L2 proteins suppresses JEV and DENV infections

**Fig. 1:**
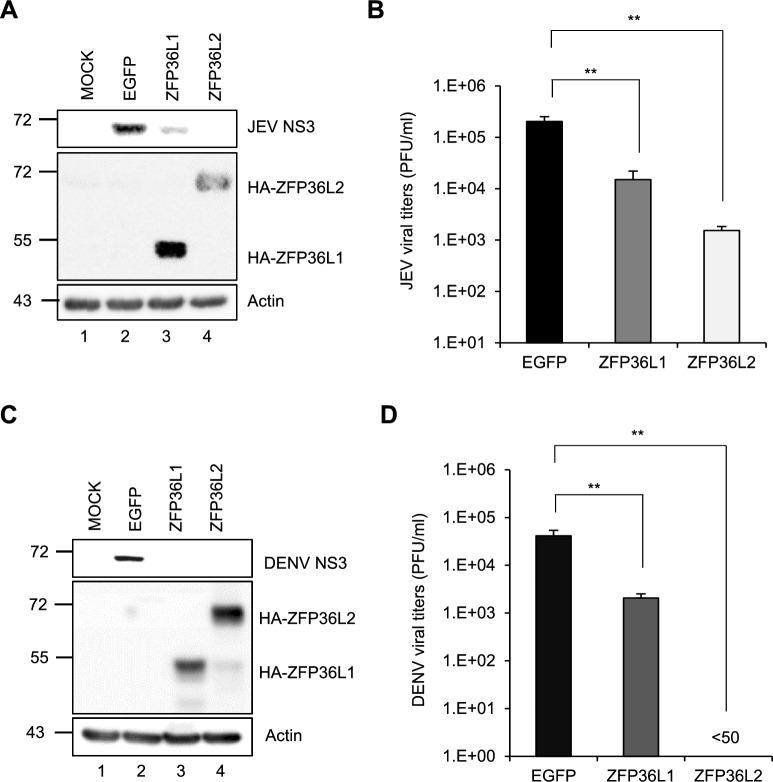
Antiviral activity of human ZFP36L2 against JEV and DENV infection. A549 cells were transduced with lentiviruses expressing EGFP, HA-tagged ZFP36L1 (HA-ZFP36L1) or HA-tagged ZFP36L2 (HA-ZFP36L2) (MOI = 2) for 72 h. Subsequently, these cells were infected with JEV **A**, **B** and DENV, **C**, **D** (MOI = 5). At 24 hpi, both cell lysates and culture supernatants were collected. **A**, **C** Cell lysates were used to determine the levels of the viral JEV or DENV NS3 protein, HA-ZFP36L2, and actin by western blot analysis. **B**, **D** Culture supernatants were used to measure viral titers by plaque assay. Representative data are presented as the mean ± SD (n = 3), and statistical significance was analyzed by two-tailed Student’s *t*-test; ** *P* ≤ 0.01

A549 cells were transduced with lentiviral vectors to overexpress HA-tagged ZFP36L1 (HA-ZFP36L1), ZFP36L2 (HA-ZFP36L2), or control EGFP ectopically, to evaluate the antiviral potential of human ZFP36-like proteins. Cells overexpressing ZFP36L1, ZFP36L2, or EGFP were infected with JEV and DENV at a high MOI, and the viral load and protein expression levels were measured at 24 h post-infection (hpi). Compared with the EGFP control, overexpression of the human ZFP36L1 and ZFP36L2 proteins decreased the level of the viral NS3 protein, as measured using western blotting (Fig. 1A, C). Moreover, the production of infectious virions was notably decreased, as assessed based on the measurement of the infectious titers using plaque-forming assays (Fig. 1B, D). The inhibitory effect of ZFP36L2 against JEV and DENV infections was also observed in biologically relevant human cell lines, including human neuroblastoma lineage SK-N-SH cells for JEV and human umbilical vein endothelial cells (HUVECs) for DENV (Fig. S1). Interestingly, ectopic expression of HA-ZFP36L1 and HA-ZFP36L2 inhibited the replication of both viruses, highlighting the potent antiviral effects of ZFP-like proteins against viral infections.

### Knockdown of the endogenous human ZFP36L2 protein enhances JEV and DENV replication

**Fig. 2:**
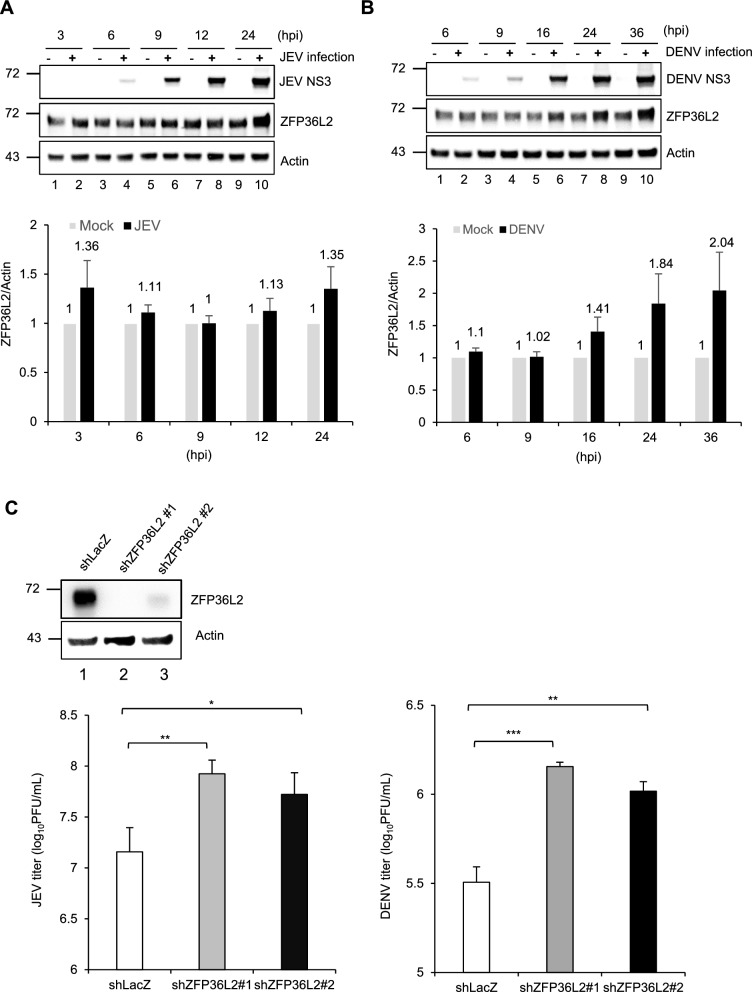
Enhancement of both JEV and DENV replication in ZFP36L2-knockdown cells. **A**, **B** A549 cells were mock infected or infected with JEV (**A**) or DENV (**B**) (MOI = 5) for the indicated periods. A Western blot analysis of the levels of ZFP36L2, viral NS3, and actin (as the loading control) is shown. The ZFP362 protein level normalized to actin was quantified using the ImageJ software. Data are representative of three independent experiments, as shown in the supplementary data (Fig. S2), and are expressed as mean ± SD (n=3). **C** A549 cells with either shLacZ or shZFP36L2 were infected with JEV or DENV (MOI = 5) for 24 h. Subsequently, the culture supernatants were used to determine the viral titer. Data from three independent experiments are presented as the mean ± SD. Statistical significance was analyzed by two-tailed Student’s *t*-test. * *P* ≤ 0.05, ** *P* ≤ 0.01, *** *P* < 0.001

To elucidate the physiological role of ZFP36L2 during flavivirus infection, A549 cells were infected with JEV and DENV at the indicated time points. Consequently, the levels of endogenous ZFP36L2 were predominantly upregulated in JEV- and DENV-infected cells, particularly during the late stages of the infection (Fig. 2A, B). Conversely, the knockdown of ZFP36L2 in A549 cells significantly increased the viral production of both JEV and DENV compared with the control cells with LacZ knockdown (Fig. 2C). These results indicated that ZFP36L2 knockdown enhances the replication of JEV and DENV, suggesting an antiviral role for ZFP36L2 in the defense of the host against flaviviruses.

### Human ZFP36L2 restricts JEV replication by destabilizing viral RNA

**Fig. 3:**
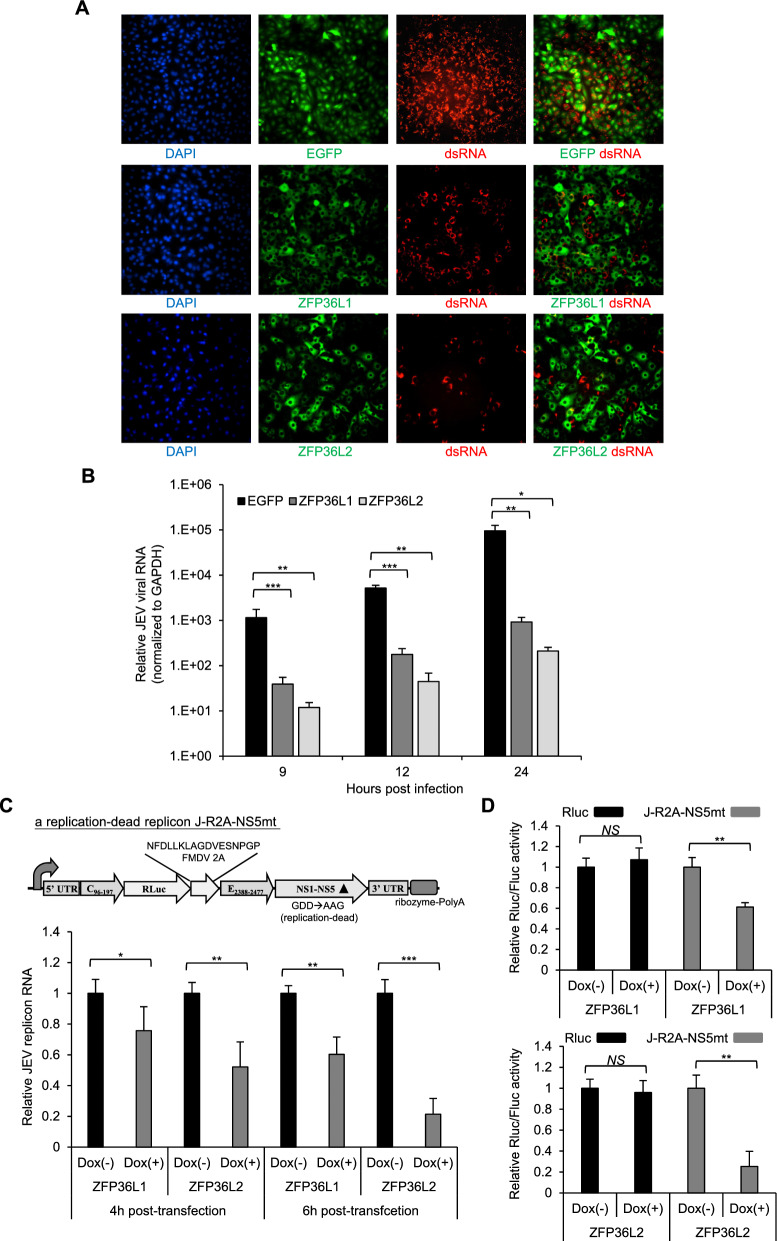
ZFP36L1 and ZFP36L2 reduce JEV viral RNA levels and inhibit JEV replication by destabilizing the viral RNA. A549 cells were transduced with lentiviruses expressing EGFP, HA-ZFP36L1, or HA-ZFP36L2 (MOI = 2) for 72 h, then infected with JEV (MOI = 5) for indirect IFA and RT-qPCR analysis. **A** At 24 hpi, indirect IFAs of cells stained with viral dsRNA (red), HA-tagged ZFP36L1 (green), HA-tagged ZFP36L1 (green), and DAPI (blue) were photographed using a fluorescence microscope. **B** RT-qPCR analysis was employed to measure the JEV RNA levels after the indicated time points. The relative JEV RNA levels were normalized to that of *GAPDH* and presented as the mean ± SD (n = 3). **C** T-REx-293 cells with inducible expression of HA-ZFP36L1 or HA-ZFP36L2 were cultured in medium without (−) or with (+) Dox (1 μg/mL) for 16 h were co-transfected with 5ʹ-capped RdRP-dead JEV replicon RNA and control firefly luciferase (Fluc) RNA. The relative RNA levels of replicon normalized to that of firefly luciferase were analyzed using RT-qPCR at 4 h and 6 h post-transfection. **D** T-REx-293 cells overexpressing HA-ZFP36L1 or HA-ZFP36L2 induced without (−) or with (+) Dox (1 μg/mL) for 16 h were co-transfected with 5ʹ-capped RdRP-dead JEV replicon RNA or 5ʹ-capped renilla luciferase (Rluc) RNA and control firefly luciferase (Fluc) RNA. The relative luciferase activity (Rluc/Fluc) was measured by dual-luciferase reporter assay at 24 h post-transfection. Data from three independent experiments are presented as the mean ± SD. Statistical significance was analyzed by two-tailed Student’s t-test. * *P* ≤ 0.05, ** *P* ≤ 0.01, *** *P* < 0.001

Our previous study demonstrated that human ZFP36L1 destabilizes viral RNA, thus interfering with replication [9]. Next, we employed a JEV model to monitor the viral RNA level in cells overexpressing ZFP36L1, ZFP36L2, and the control EGFP. The results demonstrated that cells overexpressing ZFP36L1 and ZFP36L2 exhibited significantly decreased JEV RNA levels compared with cells overexpressing the control EGFP, as evidenced by immunofluorescence assay (IFA) (Fig. 3A) and real-time quantitative polymerase chain reaction (RT-PCR) (Fig. 3B), respectively. To clarify further whether ZFP36L2 impairs JEV RNA stability, we established inducible HEK T-REx-293 cell lines expressing N-terminal HA-tagged ZFP36L1 and ZFP36L2 proteins. Western blotting revealed that the JEV NS3 protein levels were markedly decreased in T-REx-293 cells overexpressing the ZFP36L1 and ZFP36L2 proteins (Fig. S3). The in vitro-transcribed RNA transcript derived from the replication-dead JEV replicon containing the renilla luciferase reporter gene (*J-R2A-NS5mt*) was transfected into T-REx-293 cells with or without ZFP36L1 and -ZFP36L2 overexpression. The results indicated that the overexpression of ZFP36L1 and ZFP36L2 in T-REx-293 cells impacted the replicon RNA level and the expression of the luciferase reporter gene in the J-R2A-NS5mt replicon (Fig. 3C), suggesting that, similar to that observed for ZFP36L1, ZFP36L2 can destabilize the flavivirus RNA, thus interfering with viral replication.

### The zinc-finger motifs of ZFP36L2 are required for its viral RNA-binding activity and antiviral effects

**Fig. 4:**
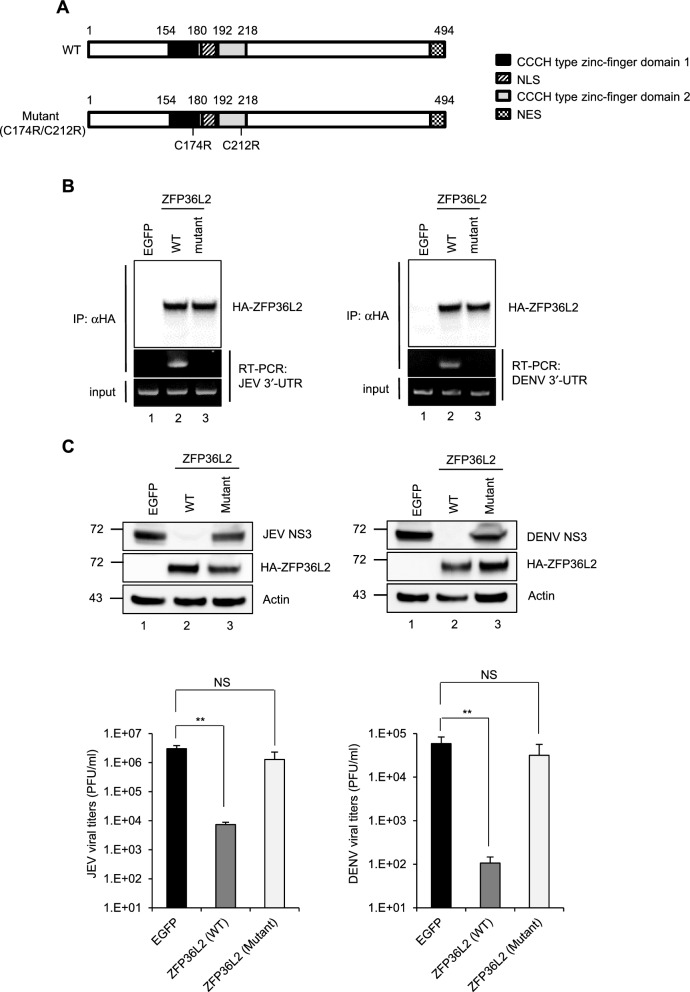
The zinc-finger motifs of ZFP36L2 are critical for its viral RNA-binding and antiviral activities. **A** Schematic representation of wild-type (WT) ZFP36L2 and the ZF domains of the ZFP36L2 (C174R/C212R) mutant. **B** 293T/17 cells were infected with JEV (MOI = 5) for 16 h or DENV (MOI = 5) for 36 h, then transfected with the indicated plasmids expressing HA-tagged ZFP36L2 (WT or C174R/C212R mutant) or EGFP for 16 h. The viral RNA bound to HA-ZFP36L2 (WT or C174R/C212R mutant) and EGFP was pulled down with anti-HA beads and amplified by RT-PCR using JEV or DENV 3′-UTR-specific primers (middle panel). Input RT-PCR used for the determination of the RNA levels of the JEV or DENV 3′ UTR in virus-infected cells (bottom panel). A western blot analysis was conducted to estimate the expression of the immunoprecipitated HA-ZFP36L2 (WT and C174R/C212R mutant) (top panel). **C** A549 cells were transduced with the lentiviral vector (MOI = 2) expressing HA-tagged ZFP36L2 (WT or C174R/C212R mutant) or EGFP for 72 h, then infected with JEV or DENV (MOI = 5) for 24 h. The culture supernatants were collected to determine the viral titers by plaque assay, and the cell lysates were examined by western blot analysis

ZFP36L2 consists of two CCCH-type ZF motifs that are conserved across the ZFP36 family and harbor RNA-binding abilities [49, 50]. To assess the importance of the ZF motifs in ZFP36L2 for viral RNA binding and antiviral effects, we generated a ZF mutant (C174R/C212R) construct (Fig. 4A). Compared with wild-type (WT) ZFP36L2, the C174R/C212R mutant did not exhibit any viral-RNA-binding ability in a radioimmunoprecipitation assay (RIPA) (Fig. 4B). Moreover, anti-HA and anti-dsRNA antibody detection combined with confocal microscopy were used to examine the cellular distribution of the ZFP36L2 proteins (WT and the C174R/C212R mutant) and viral RNA. The colocalization of WT ZFP36L2, but not of the C174R/212R mutant, with the viral RNA was primarily observed in the perinuclear region of the cytoplasm (Fig. S4). Next, we assessed the antiviral potential and observed the loss of activity of C174R/C212R against JEV and DENV using western blotting and a plaque-forming assay, respectively (Fig. 4C). The results of these experiments highlighted the role of the ZF motifs in the antiviral activity of ZFP36L2 against JEV and DENV infections.

### A divergent antiviral pathway exists between the ZFP36L1 and ZFPL2 proteins during flavivirus infection

**Fig. 5:**
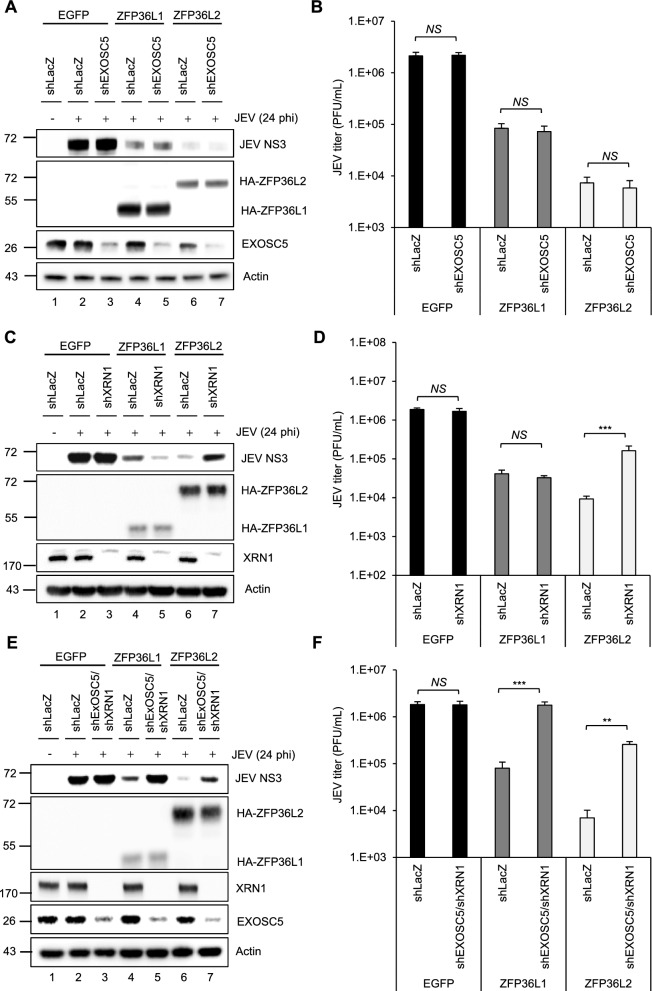
Distinct antiviral pathways employed by human ZFP36L1 and ZFP36L2 against JEV infection. A549 cells with shLacZ, shXRN1, shEXOSC5, and shXRN1/shEXOSC5 were transduced with lentiviruses expressing EGFP, HA-ZFP36L1, or HA-ZFP36L2 (MOI = 2) for 72 h. Subsequently, these cells were infected with JEV (MOI = 5). At 24 hpi, cell lysates and culture supernatants were collected. **A**, **C**, **E** Cell lysates were examined by western blot analysis of the indicated proteins. **B**, **D**, **F** Culture supernatants were used to measure the viral titers by plaque assay. Representative data are presented as the mean ± SD (n = 3), and statistical significance was analyzed by two-tailed Student’s *t*-test. ** *P* ≤ 0.01, *** *P* ≤ 0.001, NS: not significant

The proteins of the ZFP36 family interact with components of the cellular RNA decay mechanisms, including the 5′ 3′ exonuclease XRN1 and the 3′-5′ RNA exosome complex. These mechanisms facilitate the degradation of target RNAs from both the 5′ and 3′ ends, respectively [51]. In a previous study, we showed that these two cellular RNA decay pathways effectuate the antiviral activity of ZFP36L1 [9]. Therefore, we knocked down the 5′-3′ exoribonuclease (XRN1) and 3′-5′ exosome component (EXOSC5) in cells overexpressing ZFP36L1, ZFP36L2, and EGFP by lentiviral transduction, to verify whether these RNA decay machineries underlie the antiviral activity of ZP36L2. The antiviral potential of both proteins in these knockdown cells was assessed by measuring the viral NS3 protein level using western blotting and plaque-forming assays. Compared with control shLacZ cells, cells overexpressing ZFP36L1 with a double-knockdown of XRN1 and EXOSC5 (shXRN1/shEXOSC5) did not exert an anti-JEV effect (Fig. 5A–F). In contrast with the anti-JEV effect observed for ZFP36L1, ZFP36L2 lost its anti-JEV effect in the presence of XRN1 knockdown (shXRN1) and double-knockdown of XRN1 and EXOSC5 (shXRN1/shEXOSC5) (Fig. 5A–F). These results indicated that the antiviral mechanism of ZFP36L1 is effectuated through both pathways, whereas ZFP36L2 solely relies on the 5′-3′ XRN1-mediated RNA decay pathway.

### Human ZFP36L2 localization to PBs is not required for its antiviral activities

**Fig. 6:**
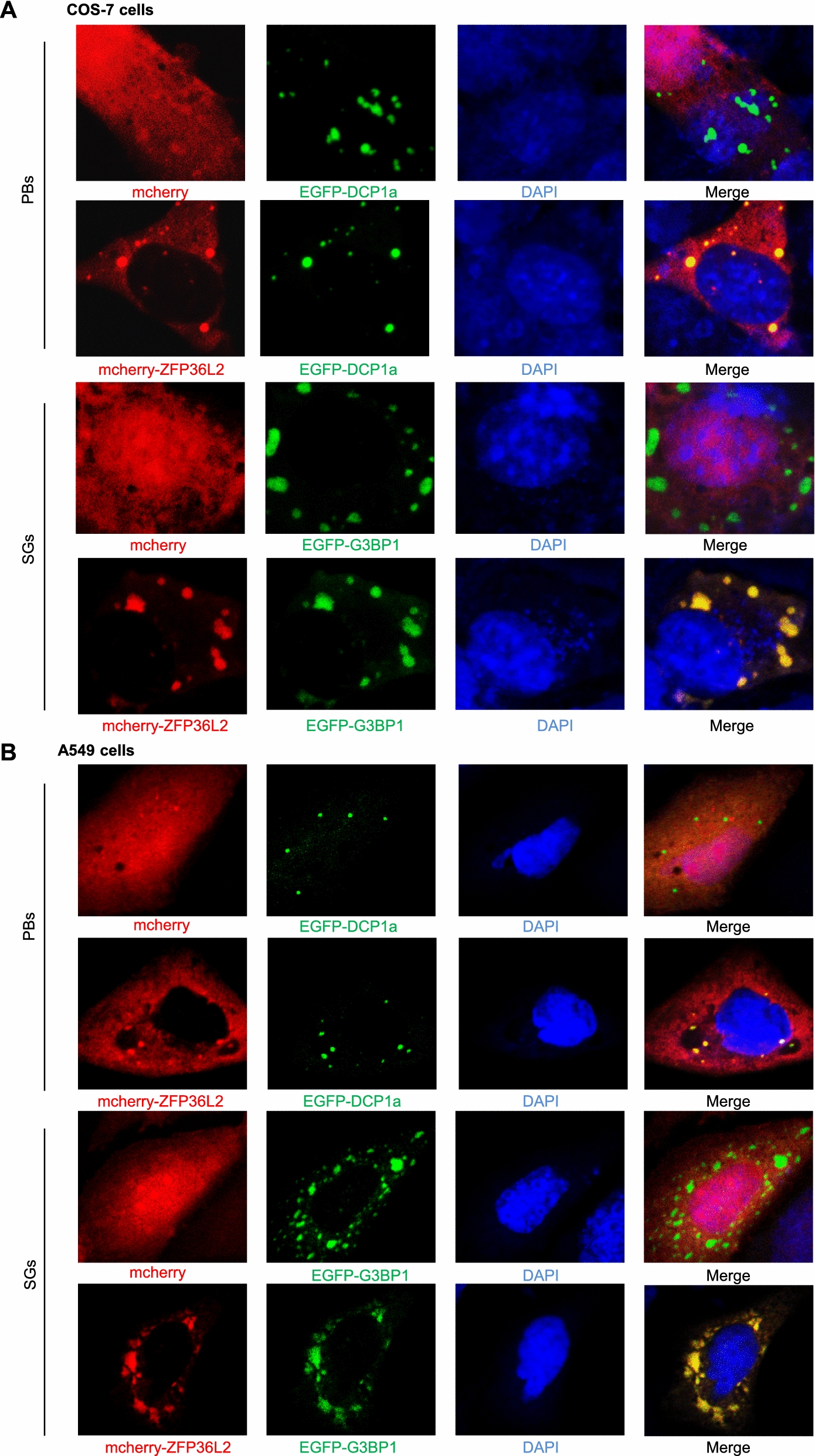
Localization of the human ZFP36L2 protein in cytoplasmic PBs and SGs. **A**, **B** A549 and COS-7 cells were co-transfected with either control mCherry (red) or mCherry-ZFP36L2 (red) plus EGFP-Dcp1a (a PB marker; green) or EGFP-G3BP1 (an SG marker; green) for 24 h. Cells were fixed and permeabilized, and the subcellular localization was analyzed by confocal microscopy

**Fig. 7:**
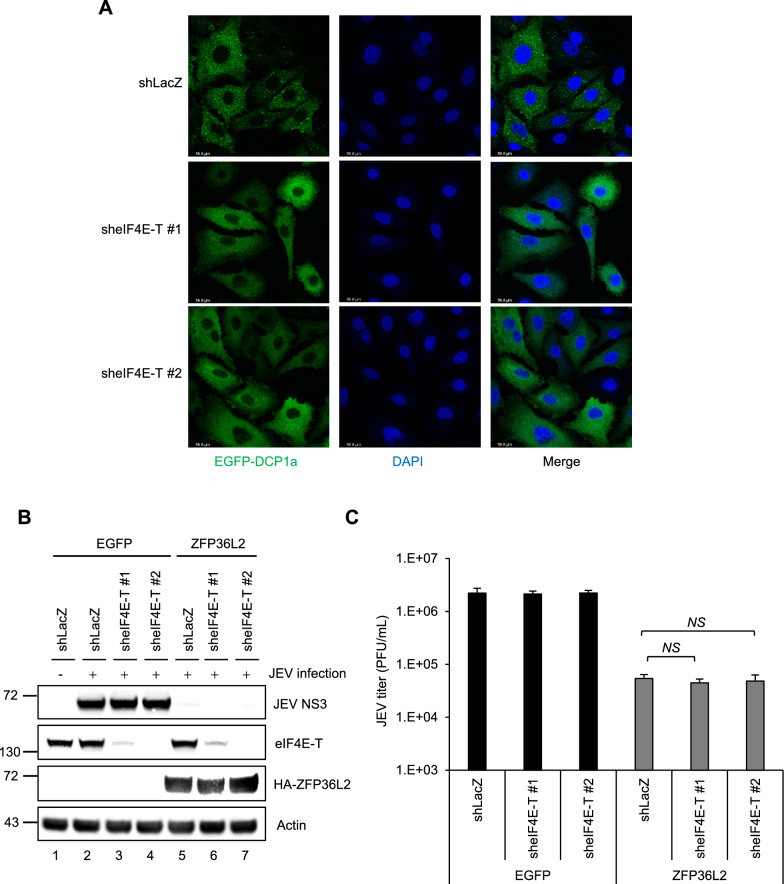
Disruption of PBs does not impact the ZFP36L2-mediated antiviral activity against JEV. **A** A549 cells with either shLacZ or sheIF4E-T were transduced with lentiviruses expressing EGFP-Dcp1a (MOI = 2) for 72 h, then treated with 0.5 mM sodium arsenite (0.5 mM) for 1 h. The formation of PBs by EGFP-Dcp1a (green) was observed using confocal microscopy. Nuclei were stained with DAPI (blue). **B**, **C** A549 cells with either shLacZ or sheIF4E-T were transduced with lentiviruses expressing EGFP or HA-ZFP36L2 (MOI = 2) for 72 h. Subsequently, these cells were infected with JEV (MOI = 5). At 24 hpi, cell lysates were harvested for western blotting using the indicated antibodies (**B**). Culture supernatants were used to measure the viral titers by plaque assay (**C**). Representative data are presented as the mean ± SD (n = 3), and statistical significance was analyzed by two-tailed Student’s *t*-test. *NS*: not significant

The TTP and ZFP36L1 proteins of the ZFP36 family are localized in SGs and PBs [52–54]. Similar to TTP and ZFP36L1, ZFP36L2 was associated with Dcp1a, which is a marker of PBs, in COS-7 cells [55]. We also observed that human ZFP36L2 colocalized with Dcp1a and G3BP1 in cytosolic granules, which are markers of both PBs and SGs, in A549 and COS-7 cells (Fig. 6A, B). This result suggests that ZFP36L2 is among the proteins that are linked to both PBs and SGs. PBs are cytoplasmic RNA granules that execute the 5′-3′ mRNA decay process via the 5′-3′ exonuclease XRN1 [32, 56, 57]. However, the correlation between the antiviral action of ZFP36L2 and its localization to PBs in virus-infected cells remains unclear. To assess whether ZFP36L2 localization to PBs is essential for its antiviral effect, we utilized lentivirus knockdown of the eukaryotic translation initiation factor 4E-transporter protein (eIF4E-T), which is required for the formation of PBs [58, 59]. After sodium arsenite treatment, the knockdown of eIF4E-T reduced the formation of cytoplasmic PBs, as evaluated by confocal imaging (Fig. 7A). The efficiency of the knockdown of endogenous eIF4E-T and the anti-JEV effect of ZFP36L2 in cells with eIF4E-T knockdown (sheIF4E-T) were validated by measuring the expression of eIF4E-T and viral NS3 proteins, respectively (Fig. 7B) using western blotting. Furthermore, the generation of infectious virions was evaluated by quantifying the infectious titers using a plaque-forming assay (Fig. 7C). Compared with the control shLacZ cells, the knockdown of eIF4E-T (sheIF4E-T) did not affect the anti-JEV activity of ZFP36L2. These results suggested that the antiviral activity of ZFP36L2 is independent of their localization to PBs.

### Subcellular colocalization of ZFP36L2, XRN1, viral RNA, and viral NS3 within the ER region yielded an antiviral action in RCs

**Fig. 8:**
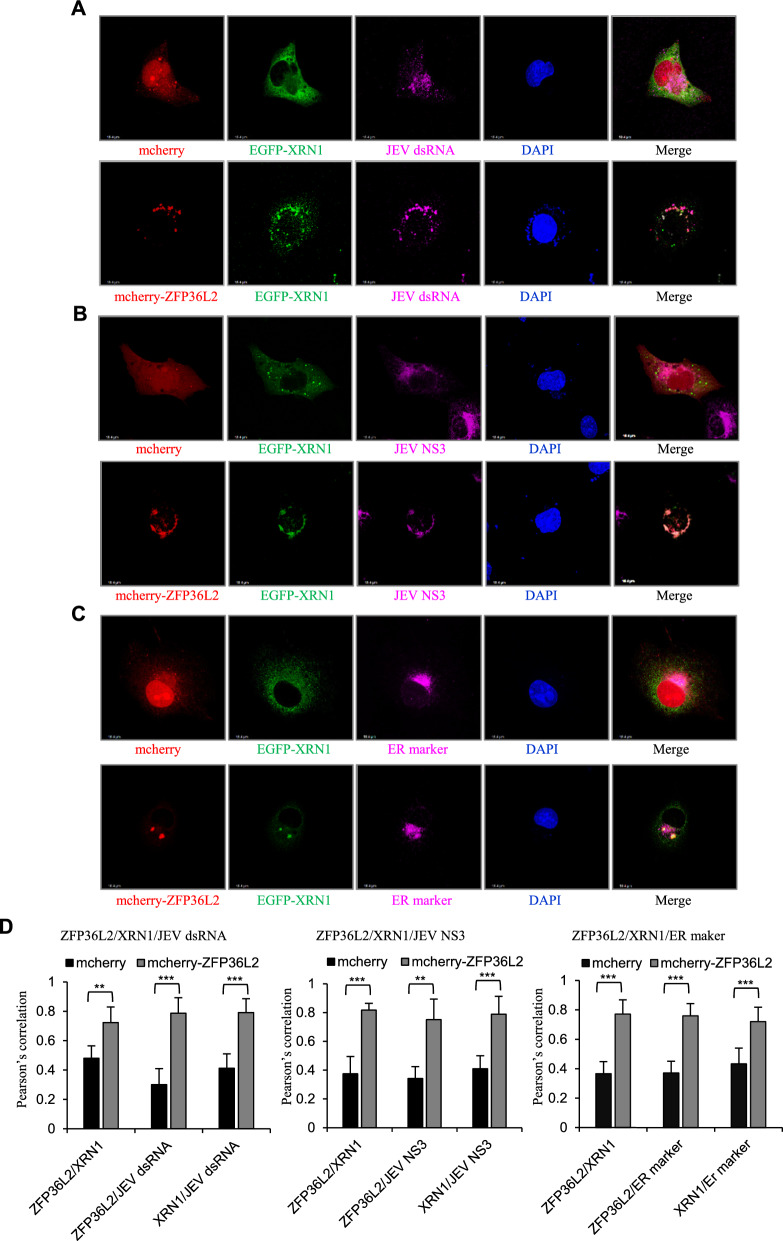
Subcellular localization of ZFP36L2, XRN1, and viral RNA in the RCs. A549 cells were infected with JEV (MOI = 5) for 3 h, then transfected with the indicated plasmids expressing either control mCherry (red) or mCherry-ZFP36L2 (red) plus EGFP-XRN1 (green) (**A**–**C**) for 16 h. Subsequently, the cells were fixed and permeabilized for confocal microscopy. **A**–**C** Viral RNA was detected using a mouse anti-dsRNA Ab and an Alexa Fluor 647 goat anti-mouse Ab (purple) (**A**). The JEV NS3 protein was detected using a mouse anti-JEV NS3 Ab and an Alexa Fluor 647 goat anti-mouse Ab (purple) (**B**). The ER was stained with an Alexa Fluo 647 anti-calreticulin Ab (purple) (**C**). Nuclei were stained with DAPI (blue). **D** Pairwise colocalization analysis was performed using Pearson’s correlation coefficient (PCC). Means and SD were calculated from 20 cells for each group. ** *P* ≤ 0.01, *** *P* < 0.001

Flaviviruses are replicated on the virus-induced intracellular membrane structures derived from the ER network. These RCs serve as platforms for viral replication by encapsulating key components of the replication complex, such as NS3 and NS5, viral RNA, and associated host factors [60–62]. Therefore, we examined the ZFP36L2-mediated antiviral effect at the RC location in JEV-infected cells. A549 cells were infected with a high MOI of JEV and subsequently transfected with either an mCherry or mCherry-fused ZFP36L2 (mCherry-ZFP36L2) construct plus an EGFP-fused XRN1 (EGFP-XRN1) construct. Compared to mCherry-overexpressing cells, mCherry-ZFP36L2-overexpressing cells retain antiviral activity as measured by immunofluorescence assay (Fig. S5). The intracellular localization of mCherry-ZFP36L2, EGFP-XRN1, and viral dsRNA was determined using an anti-dsRNA antibody and visualized using a confocal microscope. In contrast to the control mCherry, the colocalization of mCherry-ZFP36L2, EGFP-XRN1, and viral dsRNA was observed in JEV-infected cells (Fig. 8A). To confirm further the colocalization of ZFP36L2, XRN1, and viral dsRNA in the RCs, the intracellular distribution of mCherry-ZFP36L2, EGFP-XRN1, and viral NS3 was assessed using confocal imaging; the colocalization of mCherry-ZFP36L2, EGFP-XRN1, and viral NS3 was observed in JEV-infected cells (Fig. 8B). Furthermore, confocal imaging revealed the colocalization of mCherry-ZFP36L2 and EGFP-XRN1 in the ER region of JEV-infected cells (Fig. 8C). These observations were confirmed through statistical analyses of pairwise colocalization (Fig. 8D). This finding supported the theory that ZFP36L2, XRN1, and viral dsRNAs are associated with the RCs. Collectively, these results indicate that the antiviral activity of ZFP36L2 is mediated by XRN1 in the RCs.

## Discussion

Cellular RBPs are intrinsic antiviral restriction factors that act by controlling post-transcriptional RNA regulation, including the cellular mRNA decay and translation inhibition mechanisms. In previous studies, we demonstrated that ZFP36L1 inhibits influenza A virus infection by interfering with the translation of viral mRNA [63]. Moreover, ZFP36L1 targets and degrades flavivirus RNA through two distinct RNA decay pathways, i.e., 5′-3′ XRN1 and 3′-5′ RNA exosome [9]. These findings highlight the fact that human ZFP36L1, as an RBP, exerts a potent antiviral activity against influenza A virus and flaviviruses via different mechanisms, and interferes with viral RNA processes. In the present study, we identified ZFP36L2 as another member of the zinc finger 36-like protein family, which also played a role in the defense of the host against flavivirus infection, thereby expanding our understanding of the biological role of this molecule in host antiviral defense. After binding to the RNA genome of flaviviruses, the ZFP36L1 and ZFP36L2 proteins initiate viral RNA decay by recruiting and utilizing the cellular mRNA decay machinery and enzymes to degrade the target viral RNA. In contrast with the antiviral mechanism of ZFP36L1 against flaviviruses in which ZFP36L1 recruits both the 5′-3′ XRN1 and 3′-5′ RNA exosome pathways to degrade flavivirus RNA, ZFP36L2 impedes flavivirus infection through a 5′-3′ XRN1-mediated RNA decay pathway. These findings indicate that the members of the zinc finger 36-like protein family, ZFP36L and ZFP36L2, impede virus replication through distinct functions and mechanisms, especially at the posttranscriptional level.

The antiviral effect mediated by ZFP36L2 involves the cellular 5′-3′ exoribonuclease XRN1, which is a vital component of the RNA decay machinery that is responsible for executing mRNA decay starting from the 5′-end. The tight connection between the flavivirus RNA and 5′-3′ XRN1 in viral pathogenicity and the host response is well documented. XRN1 binds to flavivirus genomic RNA, initiating the degradation of the viral RNA genome through the 5′-3′ RNA decay pathway but stalls at the highly structured RNA in the 3′ UTR, producing the subgenomic flavivirus RNA (sfRNA) [64–68]. The formation and accumulation of sfRNA by XRN1 in the infected host is crucial for efficient virus replication, virus-induced cytopathicity, and viral pathogenicity [64]. Moreover, sfRNA production has multiple roles in the viral life cycle and host cells: (i) counteracting innate immune signaling, such as type-I interferon expression pathway [69–71], (ii) interfering with host mRNA homeostasis by blocking XRN1, Dicer, and other exoribonucleases [68, 72, 73], and (iii) inhibiting viral genome synthesis and translation by disrupting genome cyclization and interacting with the replication complexes [74]. Therefore, its role in generating sfRNA is significant, as sfRNA molecules interfere with host immune responses, enhance viral replication, disrupt host mRNA homeostasis, and contribute to viral pathogenicity. It will be intriguing to explore whether human ZFP 36-like proteins contribute to the generation of sfRNA molecules and viral pathogenicity during viral RNA decay, and whether sfRNA production as a negative feedback mechanism to inhibit XRN1 activity, counteracting the ZFP36L1- and ZFP36L2-mediated XRN1-dependent degradation and anti-flavivirus effects.

PBs and SGs, which are typical RNA granules in the cytoplasm, control post-transcriptional RNA regulation via cellular mRNA decay and translation inhibition, which in turn impedes virus infection [27, 28, 30, 75]. However, flaviviruses, such as WNV and Dengue virus serotype 2 (DV2), inhibit the formation of PBs and SGs by interfering with their assembly [76]. Similar to WNV and DV2, JEV has been shown to impact the formation of PBs and SGs (Fig. S6). Flaviviruses are potent inducers of cellular stress and have evolved mechanisms to subvert the formation of PBs and SGs for the host defense. Nevertheless, the disruption of PB formation through eIF4E-T knockdown did not affect the antiviral effect of ZFP36L2 (Fig. 7A–C), indicating that the ZFP36L2-mediated antiviral activity does not depend on its localization to PBs. To clarify whether ZFP36L2 localization to SGs is essential for its antiviral effect, we disrupted SGs formation by G3BP knockdown. The knockdown of G3BP, which disrupts SGs formation, did not affect the anti-JEV activity of ZFP36L2 (Fig. S7). These results indicated that the disruption of SGs and PBs does not affect the antiviral activity of ZFP36L2, suggesting that its localization to these RNA granules is not essential. In this study, we examined the distribution of ZFP36L2, XRN1, and viral dsRNA during infection, with confocal imaging revealing their colocalization within the RCs (Fig. 8A–D). These findings suggest that the effectiveness of ZFP36L2-mediated XRN1 antiviral activity against flaviviruses is not influenced by its localization to PBs and SGs; rather, the antiviral properties of ZFP36L2 are most pronounced within the RCs. A previous study reported the antiviral activity of the cellular XRN1–DCP1/2 complex and revealed that infection with RNA viruses enriches XRN1 and DCPs in viral RCs; this phenomenon is not correlated with the essential localization and function of PBs [35]. These results clarify that an XRN1-dependent antiviral activity occurs in the cytosol in viral RCs, and that its localization to PBs or SGs is not essential. ZFP36L2 restricts flavivirus infection by approximately tenfold through XRN1-mediated antiviral activity (Fig. 5D), suggesting that this pathway may partially, but not completely, accounts for ZFP36L2’s antiviral effect. Additional or distinct mechanisms may also contribute to its activity against flaviviruses, which remains to be clarified.

Proteins of the ZFP36-like family, including ZFP36L1 and ZFP36L2, are anti-inflammatory modulators that negatively regulate inflammatory cytokines and chemokines, such as the granulocyte–macrophage colony-stimulating factor (GM-CSF), TNF-α, IL-3, IL-6, and IL-8/CXCL8 [23, 77]. Patients with severe dengue disease and those with acute Japanese encephalitis exhibited excessively high levels of proinflammatory cytokines, known as a cytokine storm, including GM-CSF, TNF-α, IL-3, IL-6, and IL-8/CXCL8. [78–82]. Therefore, the ZFP36L1 and ZFP36L2 proteins may serve a dual purpose during flavivirus infection by defending against the virus and downregulating the host’s inflammatory response, to maintain a balance between the control of viral replication and the prevention of excessive inflammation. However, the actual functions of ZFP36L2 in humans, especially in controlling the viral load and preventing inflammatory illnesses, warrant further investigation. Overall, this study unveiled the crucial antiviral role of human ZFP36L2 during flavivirus infection, thus shedding light on the distinct antiviral mechanisms employed by proteins of the ZFP36-like family.

## Conclusions

However, this study highlights the observation that the antiviral function of ZFP36L2 was mediated by XRN1 within the RCs, rather than by its localization to PBs. These findings provide valuable insights into the diverse antiviral mechanisms of the ZFP36-like family that underlie the innate immune response to flavivirus infection.

## Supplementary Information

Supplementary Material 1.

## Data Availability

The data and materials supporting this article are available upon reasonable request from the corresponding author.

## Abbreviations

ZFP36L: Zinc finger protein 36-like
RBP: RNA-binding protein
JEV: Japanese encephalitis virus
DENV: Dengue virus
UTR: Untranslated region
ARE: AU-rich element
sfRNA: Small subgenomic flavivirus RNA
PBs: Processing bodies
SGs: Stress granules
RCs: Replication complexes
ER: Endoplasmic reticulum.
XRN1: 5′-3′ Exonuclease XRN1
EXOSC5: Exosome component 5
Dcp1a: MRNA-decapping enzyme 1A
G3BP1: Ras GTPase-activating protein-binding protein 1
GM-CSF: Granulocyte–macrophage colony-stimulating factor
