# Supplementary material for: The zinc finger protein ZFP36L2 inhibits flavivirus infection via the 5′-3′ XRN1-mediated RNA decay pathway in the replication complexes
Source: J Biomed Sci. 2025 Feb 20;32:27. doi: 10.1186/s12929-025-01122-0 (PMC11841009; doi:10.1186/s12929-025-01122-0)
Supplement: Supplementary file 1 — Supplementary Material 1. [file 12929_2025_1122_MOESM1_ESM.pdf]

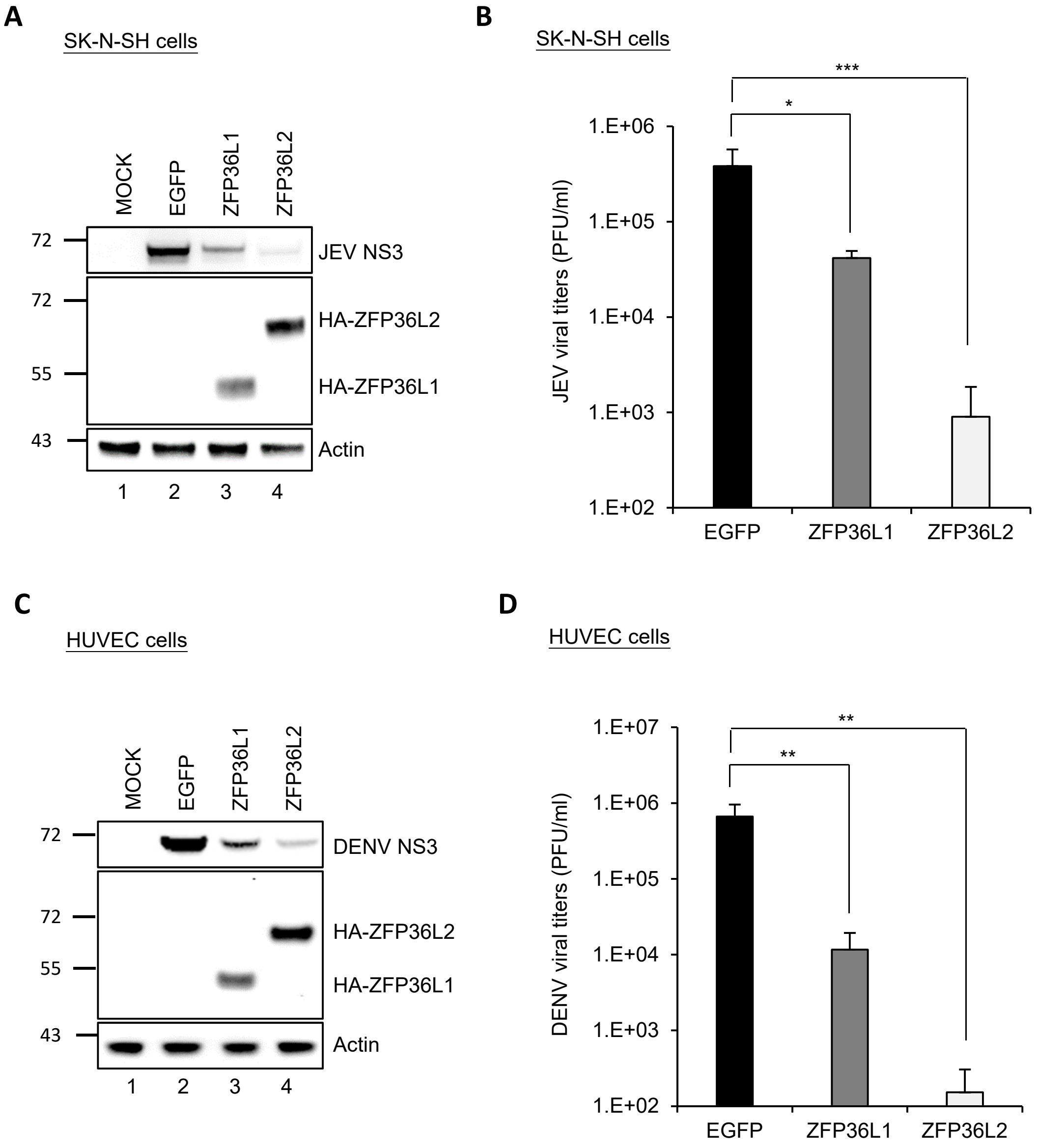

Figure S1. Overexpression of the human ZFP36L1 and ZFP36L2 proteins inhibits JEV and DENV infections in SK-N-SH and HUVEC cells, respectively.

Cells were transduced with lentiviruses expressing EGFP, HA-tagged ZFP36L1 (HA-ZFP36L1), or HA-tagged ZFP36L2 (HA-ZFP36L2) (MOI = 2) for 72 h. Subsequently, these cells were infected with JEV (**A**, **B**) and DENV (**C**, **D**) (MOI = 5). At 24 hpi, both cell lysates and culture supernatants were collected. **A, C** Cell lysates were used to determine the levels of the viral JEV or DENV NS3 protein, HA-ZFP36L2, and actin by western blot analysis. **B**, **D** Culture supernatants were used to measure the viral titers using plaque assay. Representative data are presented as the mean ± SD (n = 3), and statistical significance was analyzed by two-tailed Student's t-test; * $P \leq 0.05$, ** $P \leq 0.01$, *** $P < 0.001$.

JEV

1

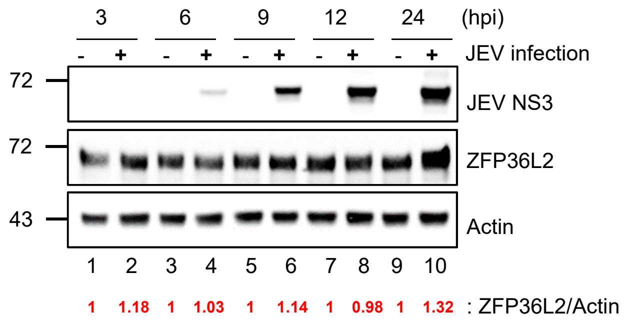

2

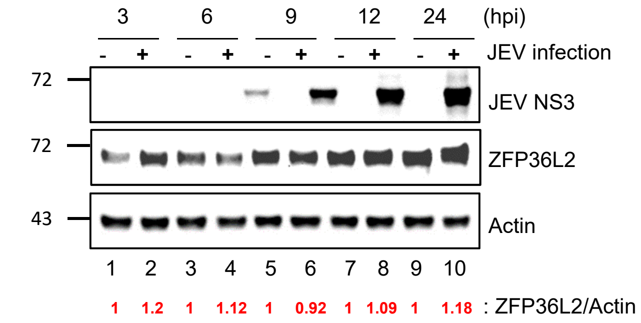

3

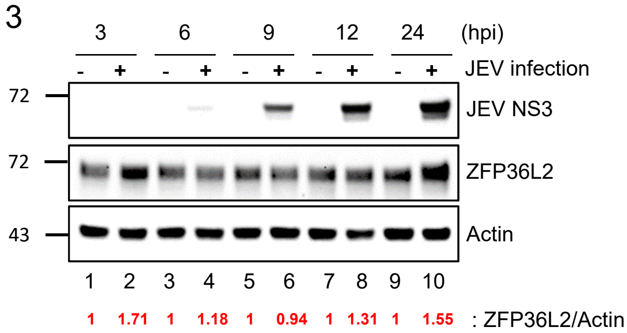

Relative intensity (ZFP36L2/Actin)

	Mock	JEV
3 phi	1	1.363333333
6 phi	1	1.11
9 phi	1	1
12 phi	1	1.126666667
24 phi	1	1.35

DENV

1

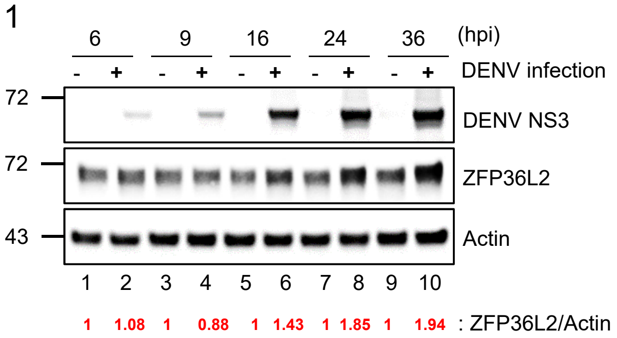

2

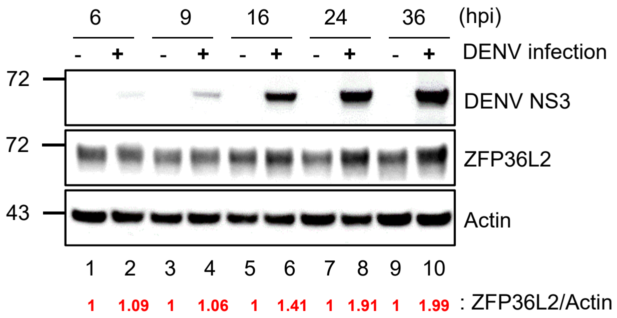

3

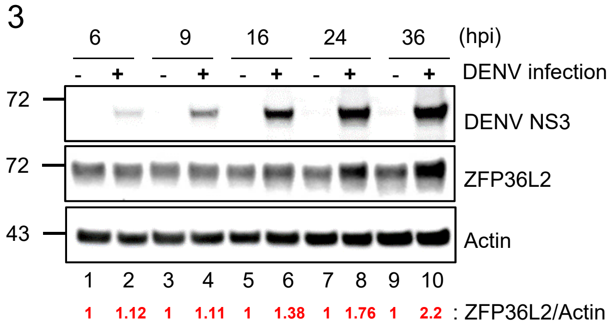

Relative intensity (ZFP36L2/Actin)

	Mock	DENV
6 phi	1	1.096666667
9 phi	1	1.016666667
16 phi	1	1.406666667
24 phi	1	1.84
36 phi	1	2.043333333

Fig. S2 The levels of endogenous ZFP36L2 in JEV- and DENV-infected cells.
A549 cells were mock infected or infected with JEV or DENV (MOI = 5) for the indicated times. Western blot analysis of protein levels of ZFP36L2, viral NS3, and actin as a loading control is shown. The relative quantification of the ZFP36L2 protein level normalized to actin level was quantified by using ImageJ. Data are representative of three independent experiments and are expressed in mean value.

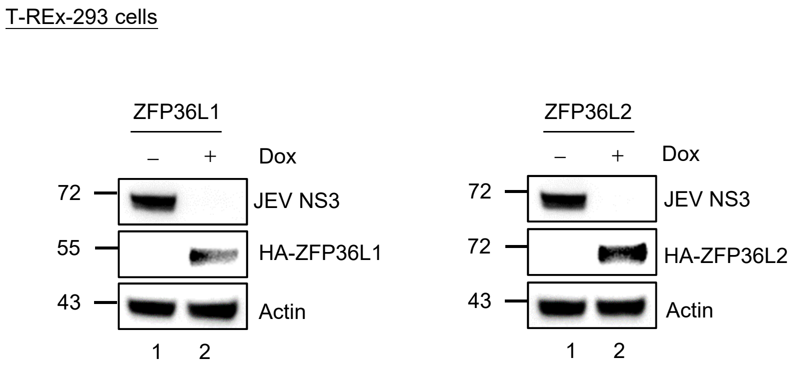

Figure S3. Overexpression of the human ZFP36L1 and ZFP36L2 proteins inhibits JEV infection in T-REx-293 cells. T-REx-293 cells overexpressing HA-ZFP36L1 or HA-ZFP36L2 were induced with or without Dox (1 µg/mL) for 16 h and then infected with JEV (MOI = 5) for 24 h. Cell lysates were examined by western blot analysis for the indicated proteins.

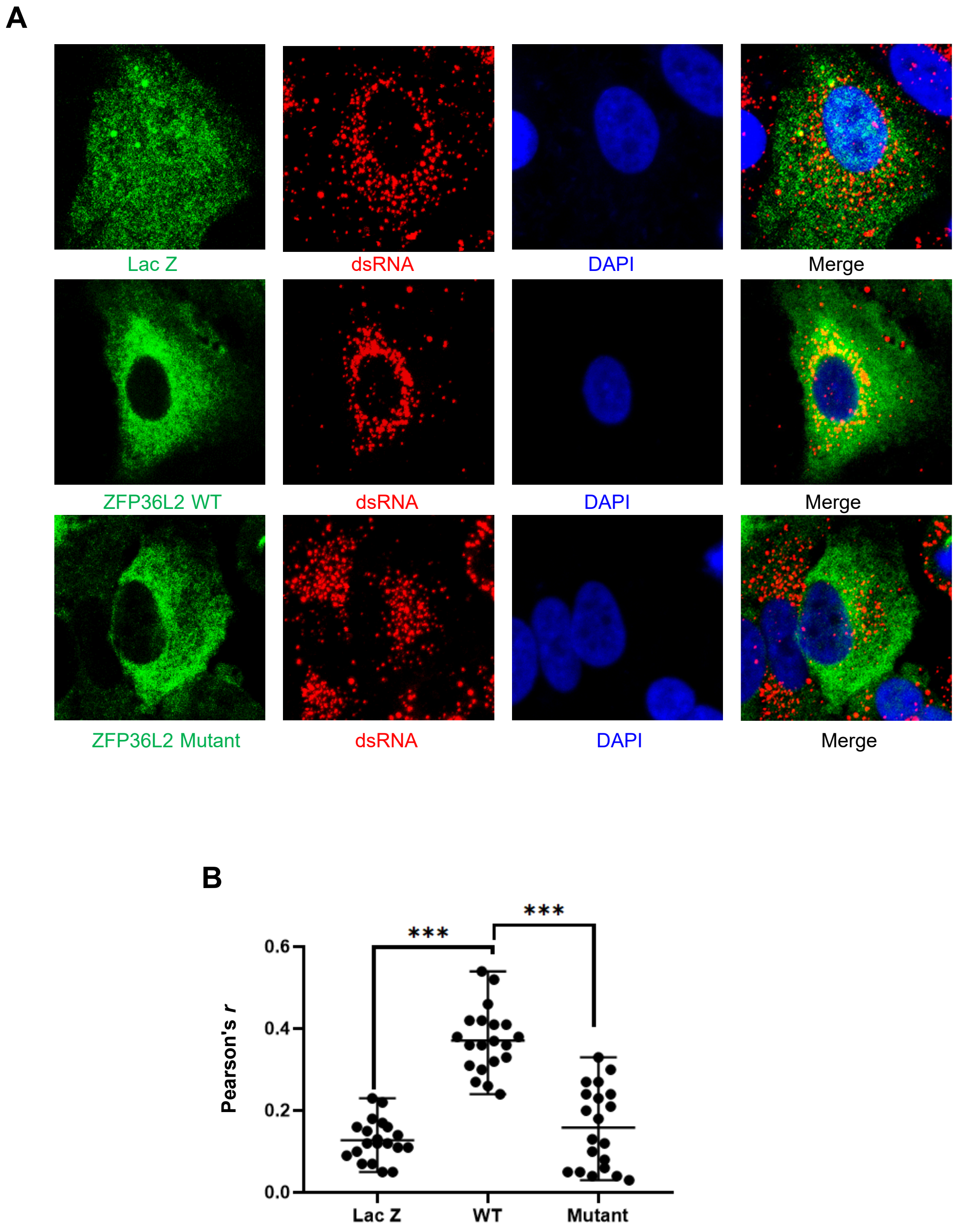

Figure S4. Subcellular colocalization of human ZFP36L2 with viral RNA.
A A549 cells were infected with JEV (MOI = 5) for 3 h and then transfected with the plasmids expressing control HA-LacZ or HA-ZFP36L2 (WT or C174R/212R mutant) for 24 h. The cellular localization of ZFP36L2 with viral RNA was analyzed by indirect IFA. Viral RNA, HA-ZFP36L2, and nuclei were detected with an anti-dsRNA Ab (red), an anti-HA Ab (green), and DAPI (blue). **B** The colocalization of HA-ZFP36L2 with dsRNA was quantified using Pearson's correlation coefficient. The mean ± SD value was calculated based on 30 cells in each group, and the statistical significance was analyzed by two-tailed Student's *t*-test. *** $P \leq 0.001$.

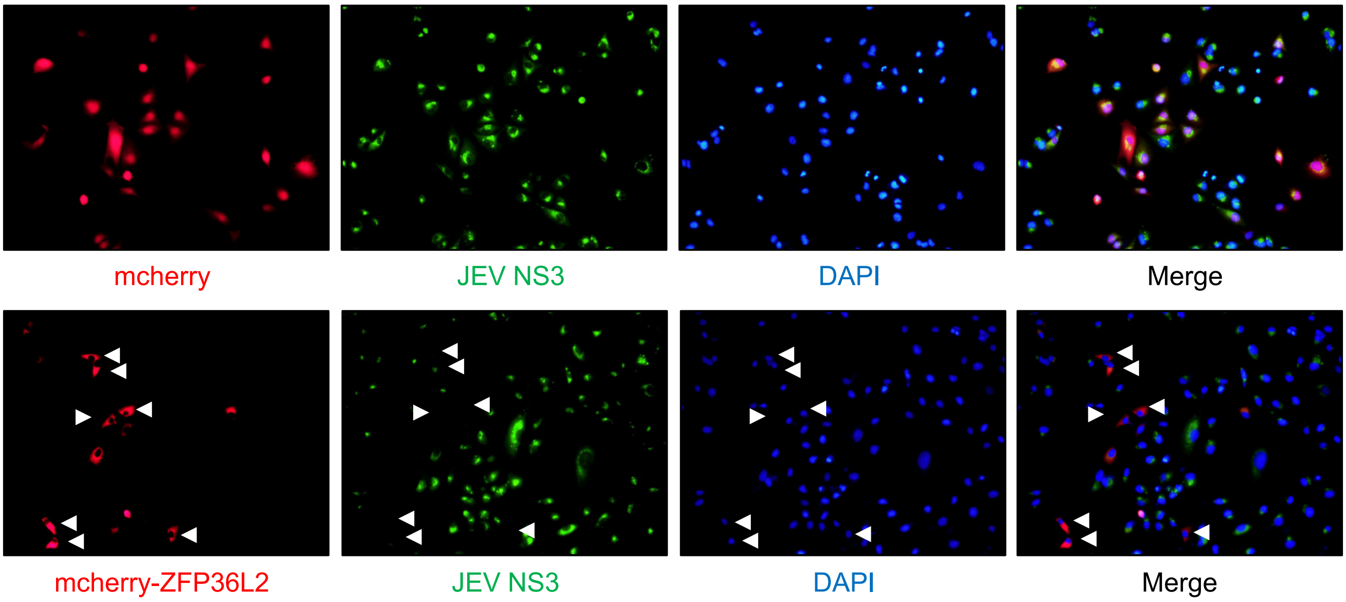

Fig. S5 Antiviral activity of mCherry-ZFP36L2 fusion protein against JEV infection.
A549 cells transduced with lentiviruses expressing mcherry or mCherry-ZFP36L2 fusion protein for 72 h were infected with JEV (MOI = 5). Cells were fixed and permeabilized for an immunofluorescent assay. At 24 hpi, cells stained with control mCherry (red) or mCherry-ZFP36L2 (red)-, viral protein NS3 (green)-, and DAPI (blue) were photographed using a fluorescence microscope. Arrows indicate the cells expressing mCherry-ZFP36L2.

A

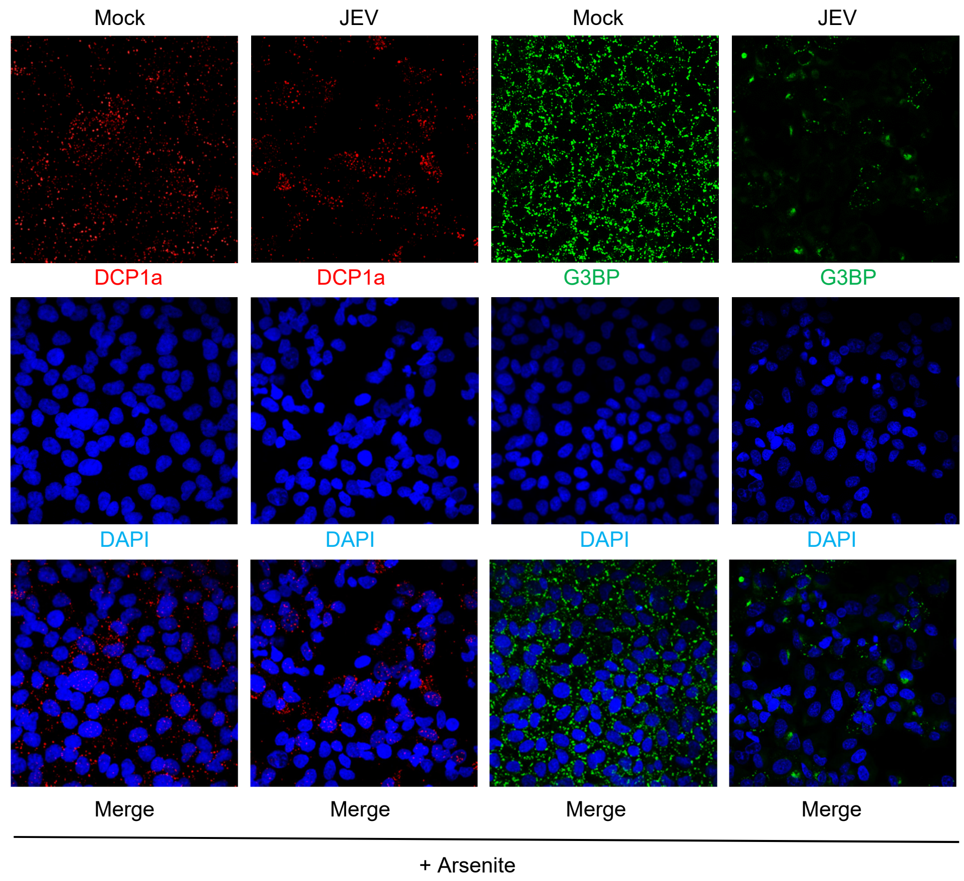

B

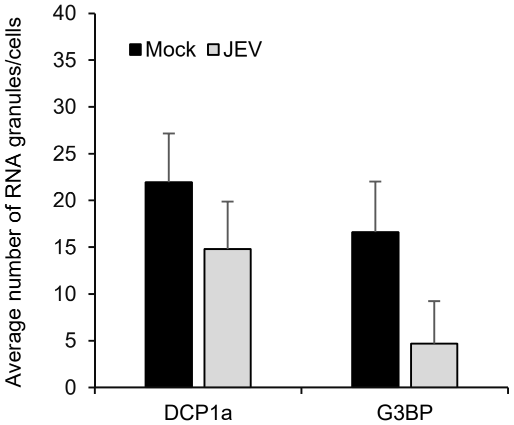

Figure S6. JEV infection disrupts the formation of cytoplasmic PBs and SGs.
A A549 cells were infected with JEV (MOI = 5) for 24 h and then treated with 0.5 mM sodium arsenite for 1 h. Subsequently, the cells were stained with anti-G3BP (green) and anti-Dcp1a (red) antibodies. Nuclei were stained with DAPI (blue). **B** The number of PBs and SGs in 40 cells was counted using the ImageJ software. The average numbers of PBs and SGs per cell were calculated for both mock and JEV-infected cells, and the values are presented as the average and the SD of the mean.

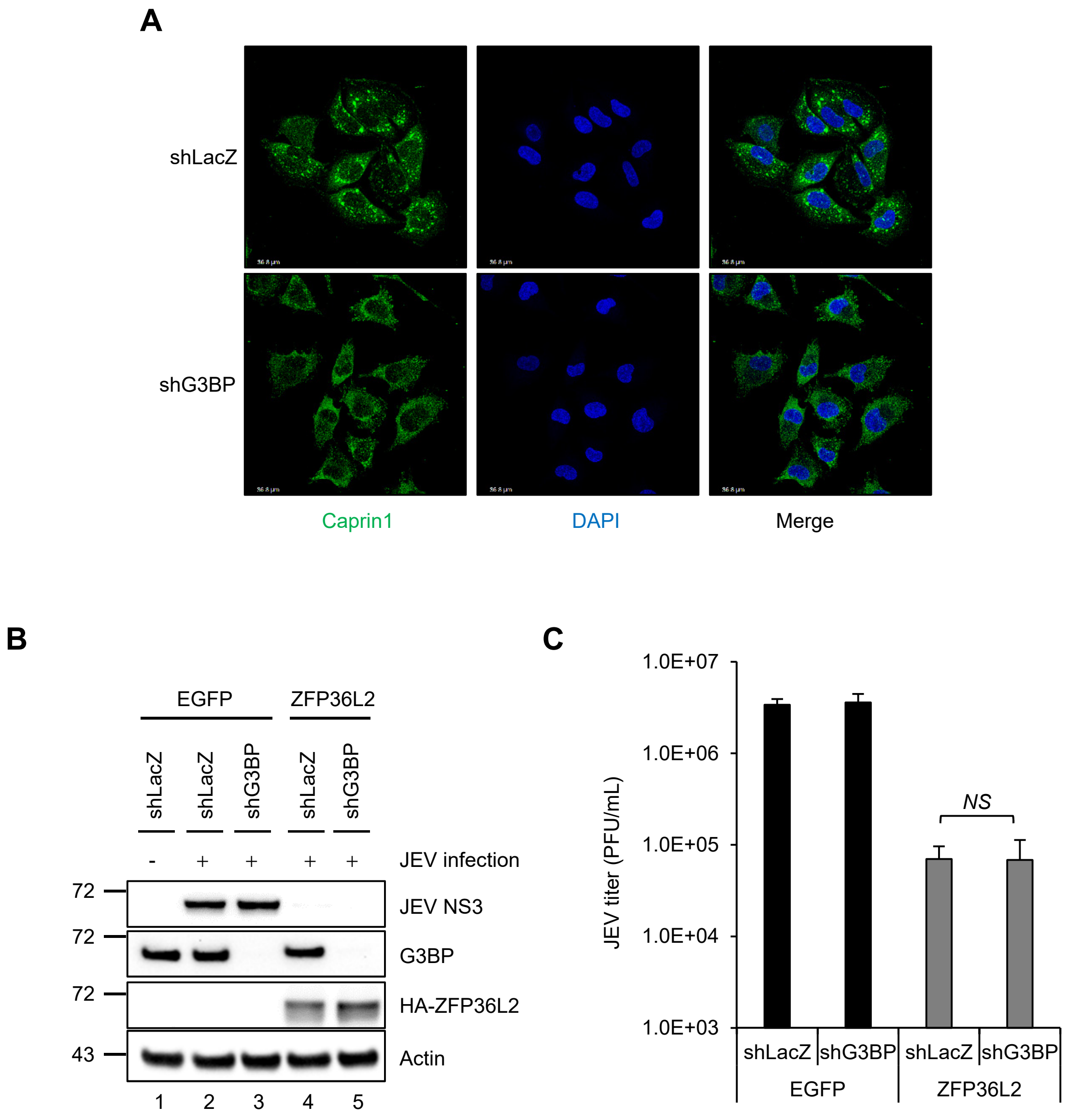

Figure S7. Disruption of SGs does not impact the ZFP36L2-mediated antiviral activity against JEV.
A A549 cells with either shLacZ or shG3BP were treated with 0.5 mM sodium arsenite (0.5 mM) for 30 min. Cells were fixed and permeabilized for confocal microscopy. The formation of SGs was detected using an anti-Caprin1 Ab (green), and the nuclei were stained with DAPI (blue). **B, C** A549 cells with either shLacZ or shG3BP were transduced with lentiviruses expressing EGFP or HA-ZFP36L2 (MOI = 2) for 72 h. Subsequently, these cells were infected with JEV (MOI = 5). At 24 hpi, cell lysates were harvested for western blotting using the indicated antibodies (**B**). Culture supernatants were used to measure the viral titers by plaque assay (**C**). Representative data are expressed as the mean ± SD (n = 3), and statistical significance was analyzed using two-tailed Student's t-test. *NS*: not significant.
